# Spoken language identification based on the enhanced self-adjusting extreme learning machine approach

**DOI:** 10.1371/journal.pone.0194770

**Published:** 2018-04-19

**Authors:** Musatafa Abbas Abbood Albadr, Sabrina Tiun, Fahad Taha AL-Dhief, Mahmoud A. M. Sammour

**Affiliations:** 1 CAIT, Faculty of Information Science and Technology, Universiti Kebangsaan Malaysia, Bangi, Selangor, Malaysia; 2 Faculty of Electrical Engineering, Department of Communication Engineering, Universiti Teknologi Malaysia, UTM Johor Bahru, Johor, Malaysia; 3 Faculty of Information and Communication Technology Universiti Teknikal Malaysia Melaka, Melaka, Malaysia; Northeast Normal University, CHINA

## Abstract

Spoken Language Identification (LID) is the process of determining and classifying natural language from a given content and dataset. Typically, data must be processed to extract useful features to perform LID. The extracting features for LID, based on literature, is a mature process where the standard features for LID have already been developed using Mel-Frequency Cepstral Coefficients (MFCC), Shifted Delta Cepstral (SDC), the Gaussian Mixture Model (GMM) and ending with the i-vector based framework. However, the process of learning based on extract features remains to be improved (i.e. optimised) to capture all embedded knowledge on the extracted features. The Extreme Learning Machine (ELM) is an effective learning model used to perform classification and regression analysis and is extremely useful to train a single hidden layer neural network. Nevertheless, the learning process of this model is not entirely effective (i.e. optimised) due to the random selection of weights within the input hidden layer. In this study, the ELM is selected as a learning model for LID based on standard feature extraction. One of the optimisation approaches of ELM, the Self-Adjusting Extreme Learning Machine (SA-ELM) is selected as the benchmark and improved by altering the selection phase of the optimisation process. The selection process is performed incorporating both the Split-Ratio and K-Tournament methods, the improved SA-ELM is named Enhanced Self-Adjusting Extreme Learning Machine (ESA-ELM). The results are generated based on LID with the datasets created from eight different languages. The results of the study showed excellent superiority relating to the performance of the Enhanced Self-Adjusting Extreme Learning Machine LID (ESA-ELM LID) compared with the SA-ELM LID, with ESA-ELM LID achieving an accuracy of 96.25%, as compared to the accuracy of SA-ELM LID of only 95.00%.

## 1. Introduction

Language Identification (LID) is the process of determining and classifying a natural spoken language from given content and datasets [1, 2]. It is undertaken by performing computational linguistics approaches and applying many contexts. These contexts include; text categorisation of a written text [3] or speech recognition of a recorded utterance [4] of a spoken identified language. It is a challenging task because due to the variations in the type of speech input and understanding how humans process and interpret speech in adverse conditions [5].

When using a LID system, several types of information are considered. Furthermore, human understanding has inspired the classification of information, and several studies have applied methods which people have used to differentiate languages, whether consciously or not. A broad classification has been used to separate or split speech features into a low level and a high level.

At the low level, most commonly used features for LID are acoustics, phonetics, phonotactics and prosodic information while at the high level, LID can be established based on the morphology and sentence syntax [6].

The acoustic features usually modelled by MFCCs are the compact representation of the input speech signal fulfilling a compression of the data contained in the audio waveform.

The phonotactic features represent admissible sound patterns formed within a given language. The N-gram language model (LM) is used to model the phonotactic features. The prosodic features refer to the duration, pitch and stress of the speech and reflect elements such as the speaker’s emotional state which cannot be characterised by the grammar used. The lexical features address the problems associated with the internal structure of words, and lastly, the syntactic features are the outcome of the analysis performed by the way in which words are linked or connected together to form phrases, clauses and sentences [6].

**Fig 1:**
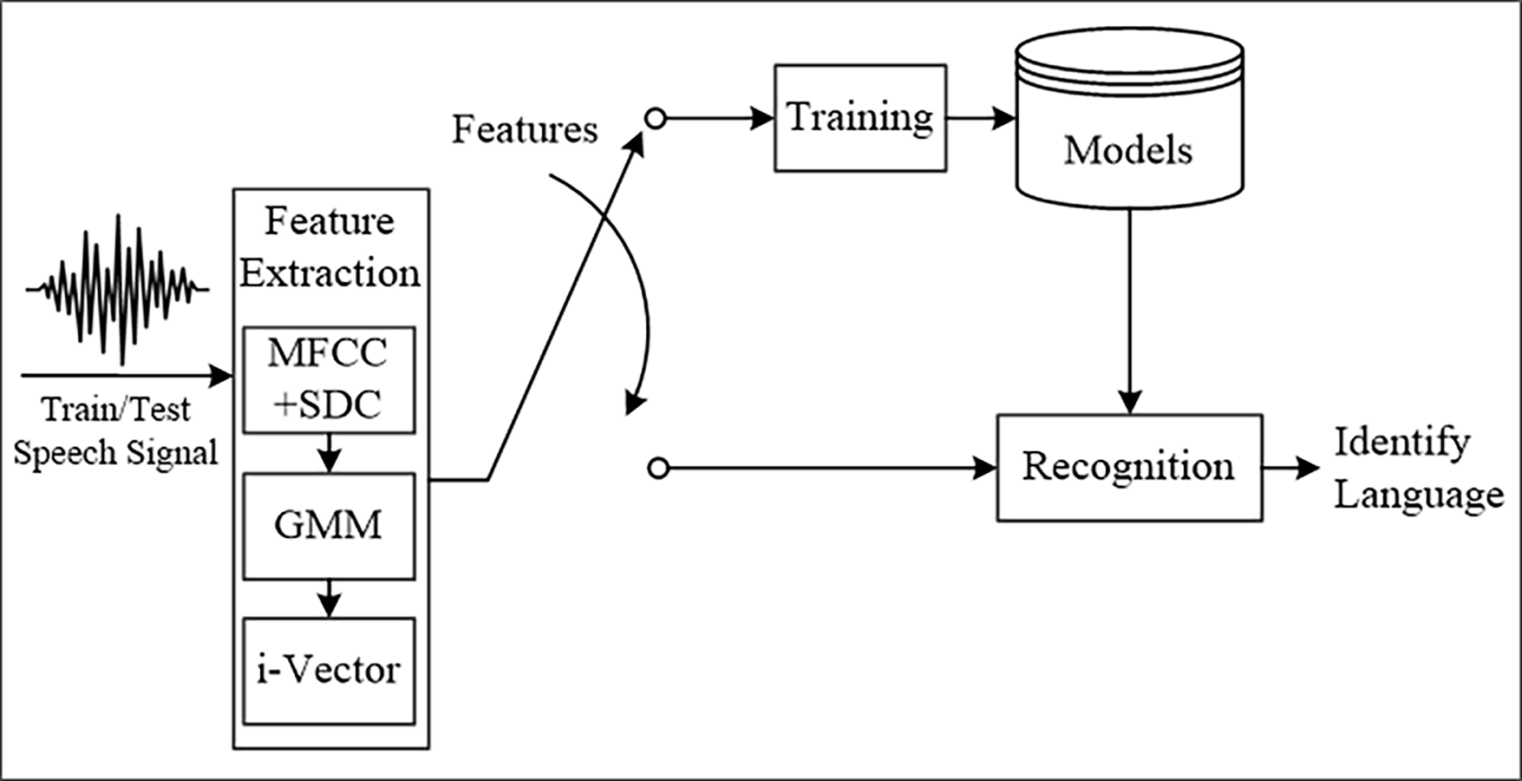
Steps of the language identification system, [6].

The conclusions, therefore, when comparing these two broad levels can be as follows. The low-level features are easier to obtain but are very volatile and are easily affected by noise and speaker variations, whereas high-level features contain more information regarding language discrimination. However, high-level features rely on large vocabulary recognisers, and as a result, more training data is needed which ultimately leads to a greater level of complexity in obtaining these features. Therefore, this study has used acoustic features and adopted the concept of feature extraction from [7] whereby the LID system is combined with a sequence of steps commencing from feature extraction, the Gaussian mixture model (GMM), i-vector construction, and recognition (classification), refer “Fig 1”.

LID is an important pre-processing technique applied to future multi-lingual speech processing systems, such as audio and video information retrieval, automatic machine translation, multi-lingual speech recognition, intelligent surveillance and so forth. A major problem in LID is how to design a specific and effective language to represent speech utterances. It is challenging due to the significant variations introduced relating to different speech patterns, speakers, channels and background noise [8]. Due to technological advances, data is being generated at an ever-increasing pace, and the size and dimensionality of the data sets continue to grow each day. Therefore, it is important to develop efficient and effective machine learning methods that can be applied to analyze the data and to extract useful knowledge and insights from the information. More recently, Extreme Learning Machines (ELMs) have emerged and have been adopted as a popular framework for machine learning [9–11]. ELMs are a type of feed-forward neural network, characterized by random initialization of their hidden layer weights, combined with a fast training algorithm. The effectiveness (i.e. without blindness) of the random initialization and fast training makes it very appealing for large data analysis.

The core classification unit is an important part of any LID system. The role of the classification unit is to map the audio sets and extract features from the i-vector system to enable its corresponding language to be identified. Different classifier types are defined in the literature such as the deep learning classifier, SVM, and ELM. ELM is described by [12], as a kind of feed-forward single hidden layer neural network, whose input weights, and thresholds of hidden layers are randomly generated. Because the output weights of the ELM are calculated utilizing the least-square method, the ELM exhibits high speed for training and testing purposes. However, the random input weights and thresholds of the hidden layers are not the best parameters, given that they cannot promise to achieve the ELM training goals and to meet global minimum requirements. The literature addresses the problem of optimizing the weights of the single-hidden layer feedforward neural networks (SLFN) trained by the ELM using various approaches. Researchers [13, 14] attempted to optimize the weights using meta-heuristic searching methods. Also, [15] aimed to optimize the weights of the ELM using the teaching phase and the learning phase under the ameliorated teaching learning-based optimisation framework. However, studies on the selection approach, to generate fresh solutions and to examine the impact on the performance of the search, are currently limited. This may lead to a slower convergence rate or incomplete optimisation. The purpose of this study is to improve the Extreme Learning Machine (ELM) algorithm by improving the self-adjusting approach and the implementation of Spoken Language Identification (LID). The final aim of the study is to prove the efficiency of the extreme learning machine as a classifier model for LID when improved optimisation is observed. The remainder of the study is organized into the following sections. Section 2 discusses related work; Section 3 describes the proposed method; Section 4 discusses and presents the experiments and results, and finally, Section 5 presents the conclusions and recommendations for future action.

## 2. Related work

The focus in this section is on machine learning and its applicability on LID as a learning model for classifying languages. The ELM is one type of classification algorithm proposed by [12] as being an effective approach towards training Single Hidden Layer Neural Network (SLNN) in one iteration. The research conducted by Huang and his team published several improvements to the extreme learning machine such as an online extreme learning machine [16] and a kernel extreme learning machine [17]. This has been proven in a wide range of applications requiring learning; human action recognition [18], Cryptography [19], image segmentation [20], face classification [21, 22], intrusion detection in cloud computing [23], Graph embedding [24], and ELMs for both semi-supervised and unsupervised tasks based on the manifold regularization [25, 26].

During the past years, extreme learning machine (ELM) [27] has been becoming an increasingly significant research topic for machine learning and artificial intelligence, due to its unique characteristics, i.e., extremely fast training, good generalization, and universal approximation/classification capability. ELM is an effective solution for the single hidden layer feedforward networks (SLFNs), and has been demonstrated to have excellent learning accuracy/speed in various applications. Thus, ELM tends to achieve faster and better generalization performance than those of back propagation (BP)-based neural networks (NNs), and SVM [27–29].

One of the important factors motivating researchers to use the extreme learning machine is its superiority over classical support vector machines from several aspects [12]. Firstly, the extreme learning machine has greater capability to avoid overfitting. Secondly, it can function on both binary and multi-type of classifiers, and thirdly, it has a neural network structure and can function as being kernel based, like SVM. All these factors add increased recognition capabilities regarding the efficiency of ELM to achieve effective learning performance.

In the field of language identification, there have been several attempts at building an ELM based language classifier to replace the classical SVM. [30] developed a new variant of an extreme learning machine applied to language identification. The improved algorithm is known as the Regularized Minimum Class Variance Extreme Learning Machine (RMCVELM). The core concept of the algorithm is to minimize the empirical risk, structural risk, and the intra-class variance. The authors evaluated it from the perspective of the execution time and level of accuracy. It outperformed SVM on the execution time and comparable classification accuracy. It is important to point out, that despite the fact of the superiority of the developed classifier, the aspect relating to the optimisation of random weights of the ELM have been ignored, causing non-optimal classification performance.

Another study applying the extreme learning machine was in the field of speaker recognition by [31]. The study used ELM on a speaker with independent text data and comparing the results with SVM. The findings from this study identified that ELM is faster to execute with much higher accuracy, however, this work is not considered as a precise application given it focused on language identification. Furthermore, their model is a binary classification model, whereby the aim of this study is to investigate using ELM in language identification, being a multi-classification problem.

A further study was conducted by [32] to identify emotions of the speaker using DNN as a feature extractor and to use extreme learning machine as a classifier. The findings identify that Kernel ELM (KELM) and ELM combined with DNN achieve the highest accuracy compared to the other baseline approaches. The authors, however, ignore the fact that ELM or KELM needs to be optimised on the input hidden layer weights.

[33] used ELM to examine the problems associated with another classifier on a different type of audio-related classification. The emotion recognition studied was based on the audio of the speaker. The features of the GMM model are used as input to the classifier with the authors emphasizing the high capabilities of GMM based features in providing a discriminative factor for classifying emotions. Unfortunately, however, minimal investigation on the effect of adding extra features to the classification or attempts to overcome the drawbacks of ELM was carried out.

## 3. Method

### 3.1 General overview

**Fig 2:**
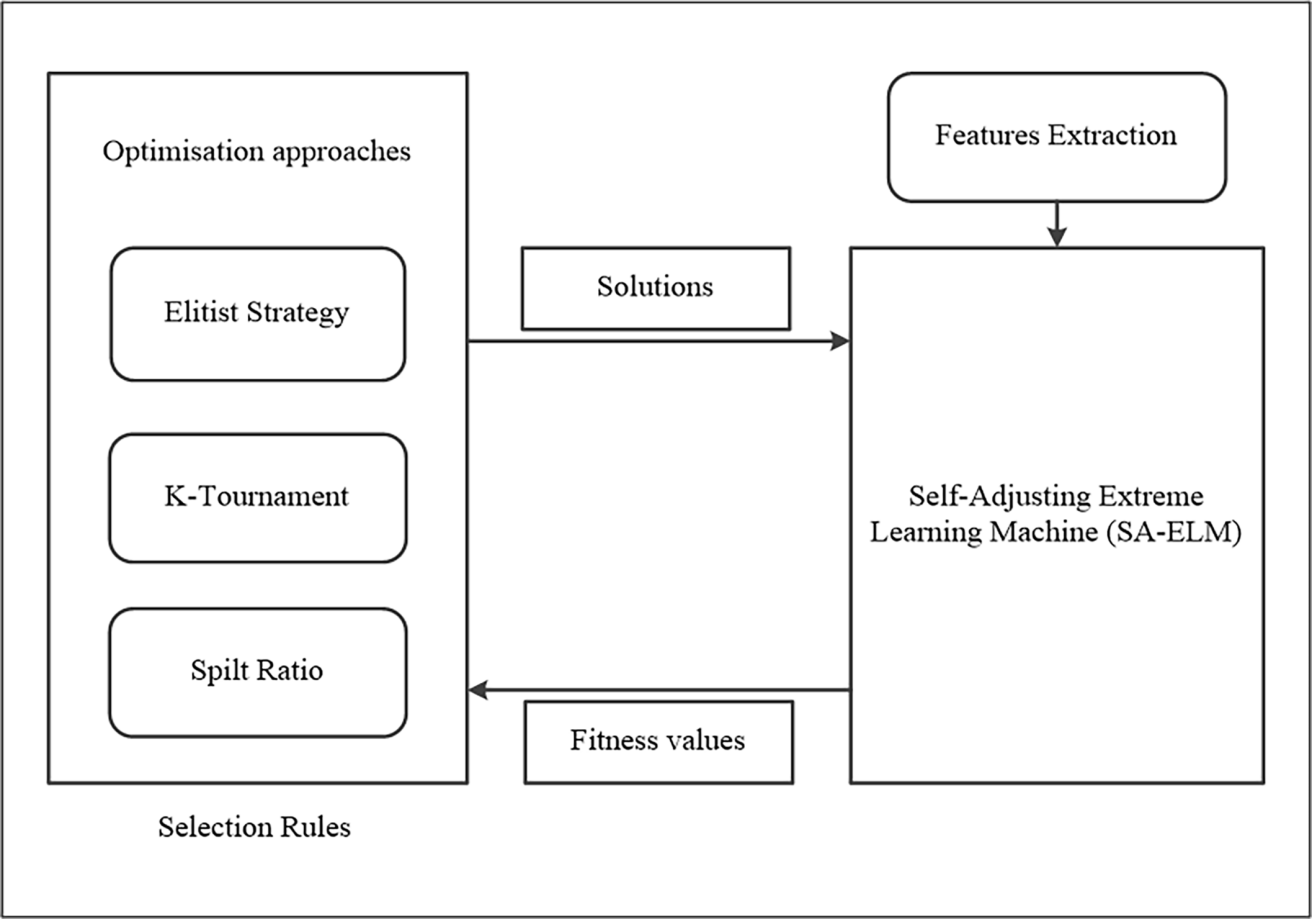
An illustrative block diagram of the optimised LID system.

The general overview of the proposed method is illustrated in “Fig 2”. The diagram shows the various blocks that will be used to create the LID system with optimised machine learning. The following sub-sections will discuss a separate area as shown in the LID system.

### 3.2 Feature extraction

The standard feature extraction for LID is adopted from [7]. Firstly, segmentation is performed to convert the input signal into frames of 25 ms with 10 ms overlap. Secondly, 7 Mel-Frequency Cepstral Coefficients (MFCCs), including C0, are obtained followed by applying Vocal Tract Length Normalization (VTLN). Next, cepstral mean and variance normalization is performed along with RASTA filtering, and this is then followed by calculating the Shifted Delta Cepstral (SDC) features in a 7-1-3-7 configuration. The results are 56-dimensional vectors consisting of both the MFCCs and the SDC. Also, GMM containing 2048 Gaussian components with diagonal covariance matrices was used with the dimensionality of the i-vectors set to 600.

### 3.3 Basic extreme learning machine (ELM)

**Fig 3:**
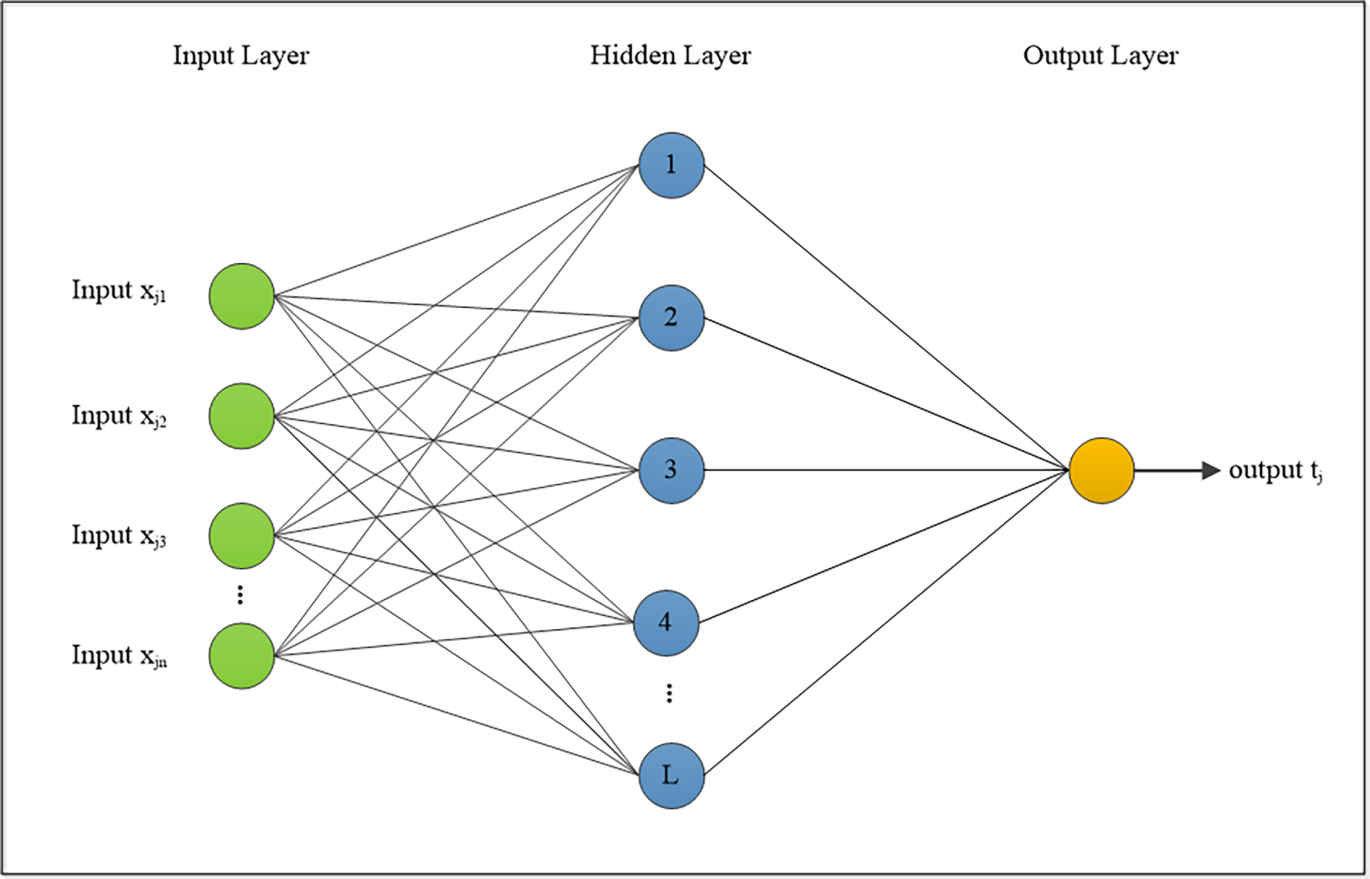
Diagram of the extreme learning machine [34].

The original ELM algorithm for training SLFN is proposed by [12]. The main concepts or ideas behind ELM are the hidden layer weights, where the biases are generated randomly. The output weights are then calculated using the least-squares solution which is defined by the outputs of the hidden layer and targets. An overview of the ELM structure and the training algorithm is shown in “Fig 3”. The next section which provides a brief description of the ELM.

Where

*N =* represents a set of distinct samples (X_i,_ t_i_), where X_i_ = [x_i1,_ x_i2…_ x_in_] ^T^ ∈ R^n^ and t_i_ = [t_i1,_ t_i2…_ t_im_] ^T^ ∈ R^m^.

*L* = indicates to the hidden layer nodes.

*g(x)* = represents the activation function, which is a mathematical model as described and applied using Eq (1)

$$\sum_{i=1}^{L}\beta_{i}g_{i}(X_{j})=\sum_{i=1}^{L}\beta_{i}g_{i}(W_{i}.X_{j}+b_{i})=o_{j}$$ (1)

*J* = 1… N.

Where:

*W*_*i*_ = *[W*_*i1*,_ *W*_*i2…*_ *W*_*in*_*]* ^*T*^ is the weight vector that provides the connection between the *ith* input nodes and the hidden node.

*β*_*i*_ = [*β*_*i*1_, *β*_*i*2_,……,*β*_*im*_] ^T^ = the weight vector that provides the connection between the *ith* output nodes and the hidden node.

*b*_*i*_ = the threshold of the *ith* hidden node.

*W*_*i*_. *X*_*j*_ = the inner product of *W*_*i*_ and *X*_*j*_. However, the output nodes are chosen linearly.

*L* = hidden nodes, and the standard of SLFNs in the activation function *g(x)* could be the samples of *N* without error.

That is:

$\sum_{j=1}^{L}\|o_{j}-t_{j}\|=0$, i.e., there exist *β*_*i*_, *W*_*i*,_ and *b*_*i*_ such that in Eq (2):

$$\sum_{i=1}^{L}\beta_{i}g_{i}(W_{i}.X_{j}+b_{i})=t_{j},\;j=1,\ldots .,N.$$ (2)

From the above equations for *N*, this can be written as follows:

Hβ=T (3)

Where:

$$H(\begin{array}{ccc} W_{1} & \ldots & W_{L,}\begin{array}{ccc} & \begin{array}{cc} b_{1} & \ldots \end{array} & b_{L,}\begin{array}{ccc} & \begin{array}{cc} X_{1} & \ldots \end{array} & X_{N} \end{array} \end{array} \end{array})$$

$$=\left[\begin{array}{ccc} g(W_{1}.X_{1}+b_{1}) & \cdots & g(W_{L}.X_{1}+b_{L})\\ \vdots & \ldots & \vdots \\ g(W_{1}.X_{N}+b_{1}) & \cdots & g(W_{L}.X_{N}+b_{L}) \end{array}\right]$$

$$\beta ={\left[\begin{array}{c} \beta_{1}^{T}\\ \beta_{L}^{T} \end{array}\right]}_{L*m}\;\mathrm{and}\;T={\left[\begin{array}{c} t_{1}^{T}\\ t_{N}^{T} \end{array}\right]}_{N*m}$$

The authors in Huang et al. (2006) named the variables, where *H* refers to the output matrix of the hidden layer in the neural network; in *H* the *ith* column refers to the *ith* hidden layer nodes on the input nodes. If the desired number of the hidden nodes is *L ≤ N*, this therefore means the activation function *g* is infinitely differentiable. Eq (3) then turns into a linear system. Furthermore, the output weights β can be determined analytically by discovering a least square solution in the following way:

$$\beta =H^{†}T$$

Where *H*^†^ is represents the Moore–Penrose generalised inverse for H. Thus, the output weights are calculated via a mathematical transformation. This makes sure that the lengthy training phrase when network parameters are iteratively adjusted with some suitable learning parameters (like iterations and learning rate) is done away with.

The authors in [12] named the variables, where *H* refers to the output matrix of the hidden layer in the neural network; in *H* the *ith* column refers to the *ith* hidden layer nodes on the input nodes. If the desired number of the hidden nodes is *L ≤ N*, this therefore means the activation function *g* is infinitely differentiable.

The weakness of ELM is that it should have a particular approach for determining the weights of the input-hidden layer weights and therefore, is subject to local minima. In other words, based on given training data, there is no way to assure that the trained ELM is the most appropriate in performing the classification. To resolve the weakness, an optimised approach must be integrated with the ELM to identify the optimal weights that assure the best performance of ELM. In the next subsection, ATLBO is presented and adopted as an optimisation approach for this very purpose.

### 3.4 Ameliorated teaching-learning-based optimisation (ATLBO)

Teaching Learning Based Optimisation (TLBO) is one of many optimisation approaches proposed by Rao et al. The algorithm has attracted many researchers’ due to its simple structure, fewer parameters and high execution speed. After developing TLBO, [35], further improvement of the algorithm was made to execute faster and to avoid selfish behaviour and presented this improvement in ATLBO.

The set of equations of ATLBO can be divided into two phases; the ‘Teaching’ phase, and the ‘Learning’ phase. The ‘Teaching’ phase means learning from the teacher, while the ‘Learning’ phase means learning through the interaction between learners. In the teaching phase, each solution is updated based on Eqs (4–6):

$$X_{new,i}=\omega_{i}X_{old,i}+\emptyset_{i}(M_{new}-T_{F}M_{i})$$ (4)

$$\omega_{i}=1/(1+exp(-fit(i)/a{p)}^{iter})$$ (5)

$$\emptyset_{i}=1/(1+exp(-fit(i)/ap)\times iter)$$ (6)

Let *Mi* = the mean, and *Ti* = Teacher (best learner) at any iteration *i*. *Ti* will try to move the mean *Mi* towards its own level, so now the new mean will be *T*_*i*_ and designated as *M*_*new*_. The solution is updated according to the difference between the existing and the new mean as depicted in Eq (4).

Where

*ω*_*i*_ = the inertia weight, which controls the effect of the former solution.

*∅*_*i*_ = the acceleration coefficient, which defines the maximum step size.

*T*_*F*_ = the teaching factor that decides the value of the mean to be changed, the value of *T*_*F*_ can be either 1 or 2.

*fit*(*i*) = the fitness of the *ith* learner.

*ap* = the maximum fitness in the first iteration.

*iter* = the current iteration.

While in the learning phase each solution is updated using Eqs (7–9).

$$X_{new,i}=\left\{ \begin{array}{c} X_{old,i}+\varphi_{i}{(X}_{j}-X_{i})\;if\;f(X_{i})\leq f(X_{j})\\ X_{old,i}+\psi_{i}{(X}_{best}-X_{i})\;if\;f(X_{i})>f(X_{j}) \end{array}\right.$$ (7)

$$\varphi_{i}=1-exp(fit{(X}_{j})-fit(X_{i}))$$ (8)

$$\psi_{i}=1-exp(fit{(X}_{best})-fit(X_{i}))$$ (9)

where

*X*_*best*_ = the best learner in a class.

*φ*_*i*_ and *ψ*_*i*_ = the acceleration coefficients that decide the step size depending on the differences between two learners.

### 3.5 Self-adjusting extreme learning machine (SA-ELM)

[15] proposed SA-ELM using the concept of an of Teaching-Learning-Based Optimisation algorithm (TLBO) consisting of two phases for adjusting the input weight and bias of hidden nodes. The first phase being the ‘teaching phase’ and the second phase being the ‘learning phase’.

The SA-ELM is described in detail as follows. The values of the input weights and thresholds of the hidden nodes are defined randomly in the teaching phase of SA-ELM, and learners' indicating the marks of all course types as shown below.

$$Vis\theta =\{\begin{array}{ccc} w_{11,} & w_{12,} & \begin{array}{ccc} \ldots & w_{1n,} & \begin{array}{ccc} w_{21,} & w_{22,} & \begin{array}{ccc} \ldots & w_{2n,} & \begin{array}{ccc} w_{m1,} & w_{m2,} & \begin{array}{ccc} \ldots & w_{mn,} & \begin{array}{ccc} b_{1,} & \ldots & b_{m} \end{array} \end{array} \end{array} \end{array} \end{array} \end{array} \end{array}\}.$$

where,

*W*_*i*j_ is the weight's value connecting between the *jth* input node and the *ith* hidden node, *W*_*ij*_ ∈ [–1, 1];

*b*_*i*_ is the bias of the *ith* hidden node, *b*_*i*_ ∈ [0, 1];

*n* is the number of input nodes; and

*m* is the number of hidden nodes.

*(n + 1) × m* represents the dimension of the learners’ mark, which means the *(n +1) × m* parameters need to be optimised. Therefore, the fitness function in the SA-ELM is set using the following Equation

$$f(\theta )=\sqrt{\frac{\sum_{j}^{N}\|\sum_{k}^{m}\rho_{k}g(w_{k}x_{j}+b_{k})-y_{j}{\|}_{2}^{2}}{N}}$$ (10)

where,

*ρ* is the output weight matrix;

y_j_ is the true value; and

N is the number of training samples.

The initial or first step calculates each target function fitness value. Following this, the learner having the minimum fitness value is selected as a teacher. The learner's new mark fundamentally relied on the previous mark *θ*_*old*,*i*_ and the difference between the former mark and the teacher (*θ*_*best*_ – *θ*_*old*,*i*_). The mechanism to update the structure of the parameters in the SA-ELM are calculated using the following Equations:

$$\theta_{new,i}=\omega_{i}\theta_{old,i}+\emptyset_{i}{(\theta }_{best}-\theta_{old,i})$$ (11)

$$\omega_{i}=1/(1+exp(-f(i)/a{)}^{iter})$$ (12)

$$\emptyset_{i}=1/(1+exp(-f(i)/a)\times iter)$$ (13)

where,

*ω*_*i*_ is the inertia weight, which controls the effect of the former mark.

*∅*_*i*_ represents the acceleration coefficient, which defines the maximum step size.

In Eqs (12) and (13), ‘a’ represents the maximum target function fitness value in the first iteration, and *iter* represents the present iteration.

Through communicating with each other, the learners increased their marks in the SA-ELM ‘learning phase’. In this step, the structure of the updated parameters used the Elitist strategy. The following Equations are used to calculate the update in the ith learner's marks, in the ith iteration.

$$\theta_{new,i}=\left\{ \begin{array}{c} \theta_{old,i}+\alpha_{i}{(\theta }_{j}-\theta_{i})\;if\;f(\theta_{i})\leq f(\theta_{j})\\ \theta_{old,i}+\beta_{i}{(\theta }_{best}-\theta_{i})\;if\;f(\theta_{i})>f(\theta_{j}) \end{array}\right.$$ (14)

$$\alpha_{i}=1-exp(f{(\theta }_{j})-f(\theta_{i}))$$ (15)

$$\beta_{i}=1-exp(f{(\theta }_{best})-f(\theta_{i}))$$ (16)

where,

*θ*_*best*_ in Eq (14), represents the best learner; *α*_*i*_ and *β*_*i*_ are acceleration coefficients which decide the step size depending on the differences between two learners.

### 3.6 Optimization approach

This section provides an explanation of the optimisation approach of the LID learning model. As previously mentioned, the ELM requires optimisation of the input hidden layer weights. The baseline approach adopts ATLBO for performing the optimisation. However, ATLBO uses only one criterion for selection. Therefore, an enhanced ATLBO or EATLBO will seek to optimize the ATLBO which is discussed further in the next sub-section along with the ESA-ELM which is based on EATLBO.

#### 3.6.1 Enhanced ATLBO (EATLBO)

The SA-ELM benchmark is based on ATLBO optimisation. The ATLBO process is divided into two parts. The first part consists of the ‘Teacher Phase’ and the second part consists of the ‘Learner Phase’. The ‘Teacher Phase’ is best described as, learning from the teacher and the ‘Learner Phase’ described as learning through the interaction between the learners. A good teacher is one who brings his or her learners up to his or her level regarding knowledge. But in practice, this is not always possible, and the teacher can only move the mean or average of a class up to some extent depending on the capability of the class. This follows a random process depending on many factors. In the ‘Learner Phase’, the Learners can increase their knowledge using two different methods. The first method is through obtaining input from the teacher, and the second method is through the interaction between them. A learner interacts randomly with other learners assisted through group discussions, presentations, formal communications, etc. A learner can learn something new if the other learner whom they are interacting with, has greater knowledge. ATLBO is based on the Elitist strategy criterion to select the best solutions in each iteration, but, this approach suffered from two problems. The first problem is that if the best solution falls into some local optima, then all other solutions will be driven towards the wrong solution and the algorithm will provide the incorrect answer. Secondly, since all solutions will follow the best solution, if there is a better solution than the one found, it may not be possible to discover. Therefore, the enhancement of ATLBO in this study, two additional criteria are incorporated, Split Ratio and K-Tournament method.

The purpose of using the k-Tournament method is to choose several solutions randomly, followed by selecting from the selected solutions the most appropriate (or best) solutions to transfer to the following generation. The Split ratio method determines how many of the identified best solutions will be transferred to the next generation, and then, from the remaining solutions, randomly selecting solutions to transfer to the next generation. Through applying this method, the search space is expanded, and the right answer is more likely to be found.

K-random samples are selected from the population to illustrate how K-Tournament works. The best solution is then selected from among the random tournament. Next, the k-Tournament is repeated until the required number of solutions is reached and then moved to the next generation. Similarly, the split ratio is applied based on a 25% - 75% ratio. This means that the algorithm will select the best 25 solutions in a deterministic manner, and then moved to the next generation while the next 75% are randomly chosen from the entire population.

#### 3.6.2 Enhanced self-adjusting extreme learning machine (ESA-ELM)

The ESA-ELM is recommended based on the concept of the Enhanced Teaching-Learning-Based Optimisation algorithm (called EATLBO). This uses the Split Ratio instead of the Elitist strategy, whose input weight values and the bias of hidden nodes are adjusted via the teaching phase and learning phase of the EATLBO. The ESA-ELM is described along with the notation of the ESA-ELM and presented in Table 1.

**Table 1:** Notation table for ESA-ELM.

Notations	Implications
**X**	Input-weight and bias assemble
*ρ*	The output weight matrix
***X***_***old*,*i***_	The previous ith solution
***X***_***new*,*i***_	The new ith solution
***X***_***best***_	The best solution
***ω***_***i***_	The inertia weight
***∅***_***i***_	The acceleration coefficient
***iter***	The current iteration
**a**	The maximum fitness value in the first iteration
***α***_***i***_	The acceleration coefficients
***β***_***i***_	The acceleration coefficients

The values of the input weights and thresholds of the hidden nodes are defined randomly in the teaching phase of the ESA-ELM and represented as learners' marks for all courses types,

$$VisX=\{\begin{array}{ccc} w_{11,} & w_{12,} & \begin{array}{ccc} \ldots & w_{1n,} & \begin{array}{ccc} w_{21,} & w_{22,} & \begin{array}{ccc} \ldots & w_{2n,} & \begin{array}{ccc} w_{m1,} & w_{m2,} & \begin{array}{ccc} \ldots & w_{mn,} & \begin{array}{ccc} b_{1,} & \ldots & b_{m} \end{array} \end{array} \end{array} \end{array} \end{array} \end{array} \end{array}\}.$$

where:

*W*_*ij*_ is the weight's value connecting between the *jth* input node and the *ith* hidden node, *W*_*ij*_ ∈ [–1, 1];

b_i_ is the bias of the *ith* hidden node, b_i_ ∈ [0, 1];

*n* is the number of input nodes; and

*m* is the number of hidden nodes.

*(n + 1) × m* represents the dimension of learners’ mark, which therefore requires the *(n +1) × m* parameters to be optimised. Therefore, the fitness function in the ESA-ELM set is calculated using the following Equation

$$f(X)=\sqrt{\frac{\sum_{j}^{N}||\sum_{k}^{m}\rho_{k}g(w_{k}x_{j}+b_{k})-y_{j}{\left|\right|}_{2}^{2}}{N}}$$ (17)

where,

*ρ* is the output weight matrix;

y_j_ is the true value; and

*N* is the number of training samples.

In the first step, the target function fitness value is calculated. Then, the learner having the minimum or lowest fitness value is chosen as a teacher. The learner's new mark fundamentally relied on the previous mark X_old,i_ and the differences between the former mark and the teacher *(X*_*best*_ – *X*_*old*,*i*_*)*. The mechanism to update the structure of the parameters in the ESA-ELM is calculated using the following Equations

$$X_{new,i}=\omega_{i}X_{old,i}+\emptyset_{i}{(X}_{best}-X_{old,i})$$ (18)

$$\omega_{i}=1/(1+exp(-f(i)/a{)}^{iter})$$ (19)

$$\emptyset_{i}=1/(1+exp(-f(i)/a)\times iter)$$ (20)

where,

*ω*_*i*_ is the inertia weight, which controls the effect of the former mark; and

*∅*_*i*_ representing the acceleration coefficient, defining the maximum step size.

In Eqs (19) and (20), ‘a’ represents the maximum target function fitness value in the first iteration, and *iter* represents the present iteration.

Through communicating with others, the learners improved their marks in the ‘Learning Phase’ of ESA-ELM. In this step, the mechanism to update the structure of the parameters, adopted the Split Ratio method to calculate the ith learner's marks in the ith iteration, as shown in the following Equations

$$X_{new,i}=\left\{ \begin{array}{c} X_{old,i}+\beta_{i}(X_{j}-X_{i})\;if\;f(X_{i})\leq f(X_{j})\\ X_{old,i}+\alpha_{i}{(X}_{best}-X_{i})\;if\;f(X_{i})>f(X_{j}) \end{array}\right.$$ (21)

$$\alpha_{i}=1-exp(f{(X}_{j})-f(X_{i}))$$ (22)

$$\beta_{i}=1-exp(f{(X}_{best})-f(X_{i}))$$ (23)

Where, Eq (21), *X*_*best*_ represents the best learner; *α*_*i*_ and *β*_*i*_ are the acceleration coefficients which decide the step size depending on the differences between two learners. The learning algorithm of the ESA-ELM performed using the following steps:

- Step (1): Generate the input weights and the bias of the hidden layer (i.e. a number of students) randomly which sets the population number and target function.
- Step (2): ‘Teaching phase’, calculates the fitness value, thereby updating the structure parameters applying Eq (18).
- Step (3): ‘Learning phase’, adopts the Split Ratio method to update the parameters using Eq (21).

**Fig 4:**
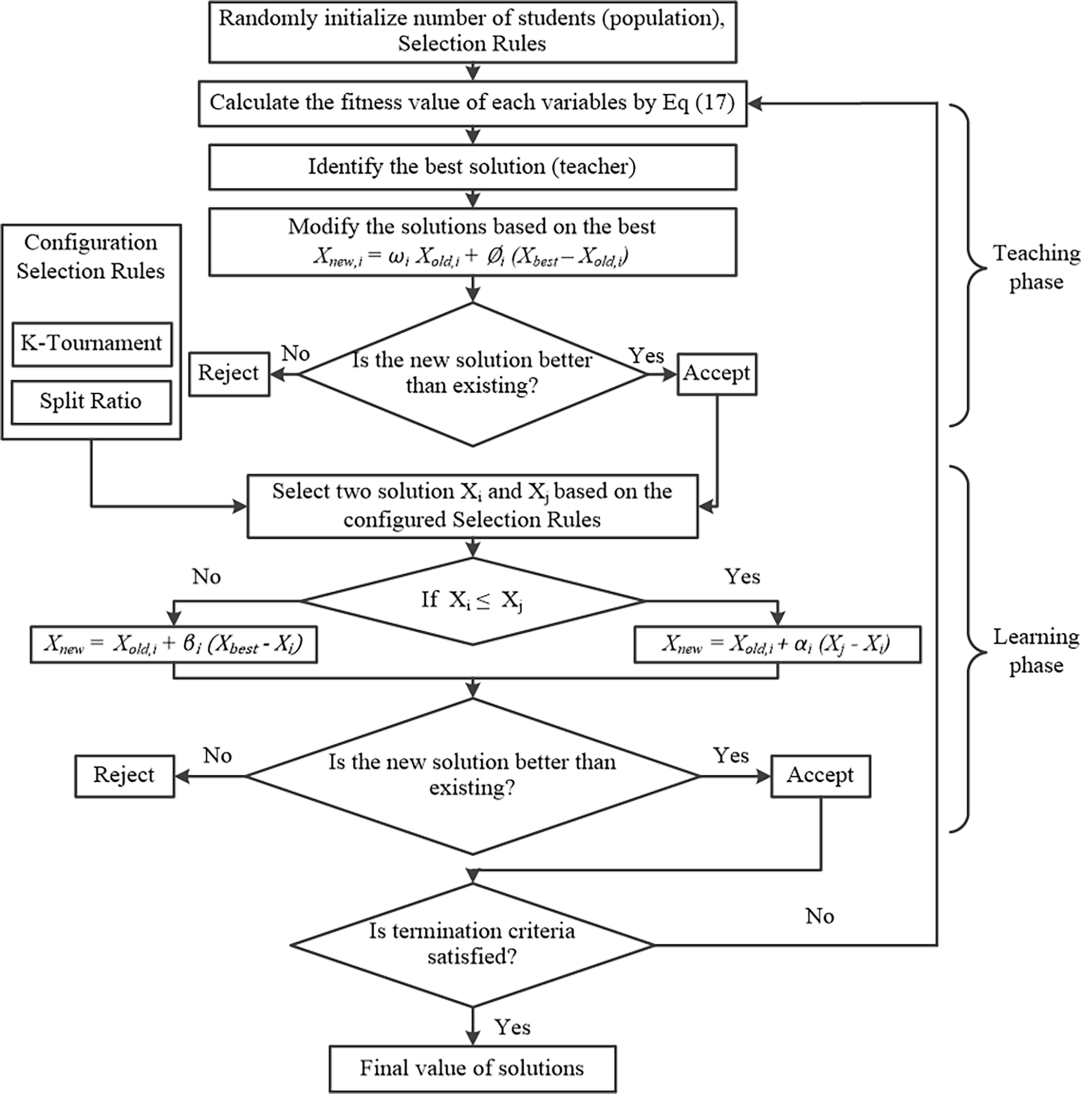
Flowchart illustrating the ESA-ELM algorithm.

According to explanations noted above, regarding the ESA-ELM, this can be described further with the aid of a flowchart illustrating the ESA-ELM algorithm and steps. “Fig 4”. represents the flowchart of the ESA-ELM algorithm.

## 4. Experiments and results

### 4.1 Raw dataset preparation

Eight different spoken languages were selected and tested for recognition purposes. The languages were; 1) Arabic, 2) English, 3) Malay, 4) French, 5) Spanish, 6) German, 7) Persian, and 8) Urdu with audio files recorded from broadcasting media channels in those respective countries. The following media broadcasting channels were:

1. Arabic: Syrian broadcast TV;
2. English (British): British Broadcasting Corporation (BBC);
3. Malay: TV9, TV2, TV3;
4. French: TF1 HD;
5. Spanish: Real Madrid TV HD, La1, La2;
6. German: Zweites Deutsches Fernsehen (ZTV);
7. Persian: Islamic Republic of Iran News Network (IRINN); and
8. Urdu: GEO Kahani.

Each language consisted of 15 utterances, with the duration of each utterance recorded being 30 Sec. 67% of the datasets were used for training, and 33% of the datasets were used for testing purposes. The audio files were recorded from respective channels as mentioned, with each dataset representing a different language to test the robustness of the algorithm.

All utterances were recorded using an mp3 format with a dual channel, using MATLAB as an array consisting of two similar columns although, only one column was used. The utterance term was the equivalent to one vector of the sampled data from the audio file. Each utterance was 30 seconds in length and required to be sampled and quantised:

1. Sampling rate: (44100 Hz), the largest frequency was (22050 Hz) referencing the Nyquist frequency. The 30 seconds’ length was approximately (30 * 44100 = 1323000).
2. Quantisation: representing real-valued numbers as integers using a 16-bit range (with values from -32768 to 32767).

The dataset that has used is described in the following below:

- Dataset name (with extension): iVectors.mat.
- Dataset dimensions as presented in Table 2:
- Class description as provided in Table 3:
- Features description as depicted in Table 4:
- Class-label-column number: Last column (601)

**Table 2:** Dataset dimension.

Number of records	Number of classes	Number of features
**120**	8	600

**Table 3:** Class description.

Number	Meaning	Number of records
**1**	Arabic	15
**2**	English	15
**3**	Malay	15
**4**	French	15
**5**	Spanish	15
**6**	German	15
**7**	Persian	15
**8**	Urdu	15

**Table 4:** Features description.

Number	Name	Type
**1 → 600**	i-vector values	Single

### 4.2 Evaluation scenario

This section discusses the evaluation measures of the EATLBO and ESA-ELM. Firstly, the EATLBO was compared with the original ATLBO for several standard mathematical functions relating to the optimisation surface. Secondly, the ESA-ELM was evaluated on several different parameters of the learning model.

#### 4.2.1 Evaluation of common mathematical functions

Five experiments applying five different objective functions were conducted for ATLBO and the EATLBO (k-Tournament and Split Ratio), with the number of iterations equivalent to 1000. The purpose of using five different objective functions was to evaluate the performance of choosing the optimal (i.e. best) fitness value for the ATLBO and the EATLBO (k-Tournament and Split Ratio) in all iterations. Table 5, represents the fitness values obtained from the ATLBO and the EATLBO (k-Tournament and Split Ratio).

**Table 5:** Testing results of optimizing benchmark mathematical functions.

Function number	Function Name	Number of variables	EATLBO (Split Ratio) fitness	EATLBO (K-Tournament) fitness	ATLBO fitness	Optimum value
**1**	Ackley's	10	0	0	0.3445	0
**2**	Alpine #2	10	-26454	-370.3588	-119.7927	-30491
**3**	Styblinski Tang	10	-320.8240	-262.8895	-236.5990	-391.6620
**4**	Egg-Holder	2	-838.5126	-759.1134	-759.1971	-959.6407
**5**	Deb's No.01	10	-1	-0.8806	-0.8180	-1

Comparing EATLBO and ATLBO it can conclude that the former has outperformed the latter. However EATLBO in this comparison is based on K-Tournament which might not be the best. Thus another selection criteria will be investigate. Therefore, another method called the Split Ratio method was also used. The results as shown in Table 1, illustrate the EATLBO (split Ratio) providing a fitness value closer to the optimal value, meaning that the performance of the EATLBO (split Ratio) was better compared to both the EATLBO (K-Tournament) and the ATLBO.

#### 4.2.2 Evaluation on different learning model parameters

Several classification experiments were conducted on the formulated datasets with both the SA-ELM benchmark and the ESA-ELM (Split Ratio) method, varying the number of hidden neurones in the range [650–900] with an increment or step of 25. Therefore, the number of all experiments for the SA-ELM benchmark was 11, and similar for ESA-ELM (Split Ratio) and the number of iterations for each test was equal to 500 iterations. The Split ratio method was selected to generate the remaining results due to its advantages over using the K-tournament method.

The evaluation performed in this study is based on [36] which presents different measures applied for the evaluation. This article was selected because it addresses the problem of classifier evaluation, and provides effective measures. Supervised Machine Learning (SML) has several ways to evaluate the performance of learning algorithms and produced classifiers. Measures relating to the quality of the classification are created from a confusion matrix which records recognised examples for each class based on their correction rate.

In this study, several evaluation measures were used to evaluate the SA-ELM (benchmark) and the ESA-ELM (split ratio) based on the ground truth. Furthermore, the evaluation measures have been adopted to compare the benchmark with the ESA-ELM (split ratio) regarding true positive, true negative, false positive, false negative, accuracy, precision, recall, F-measure and G-mean. The evaluation measures used in this study are depicted in Eqs (24–28)

$$accuracy=\frac{tp+tn}{tp+tn+fn+fp}$$ (24)

$$precision=\frac{tp}{tp+fp}$$ (25)

$$recall=\frac{tp}{tp+fn}$$ (26)

$$F-Measure=\frac{\left(2\times precision\times recall\right)}{\left(precision+recall\right)}$$ (27)

$$G-Mean=\sqrt[{2}]{\frac{tp}{p}\times \frac{tn}{n}}$$ (28)

where:

*tp* = true positive, *tn* = true negative, *fp* = false positive, and *fn* = false negative.

**Fig 5:**
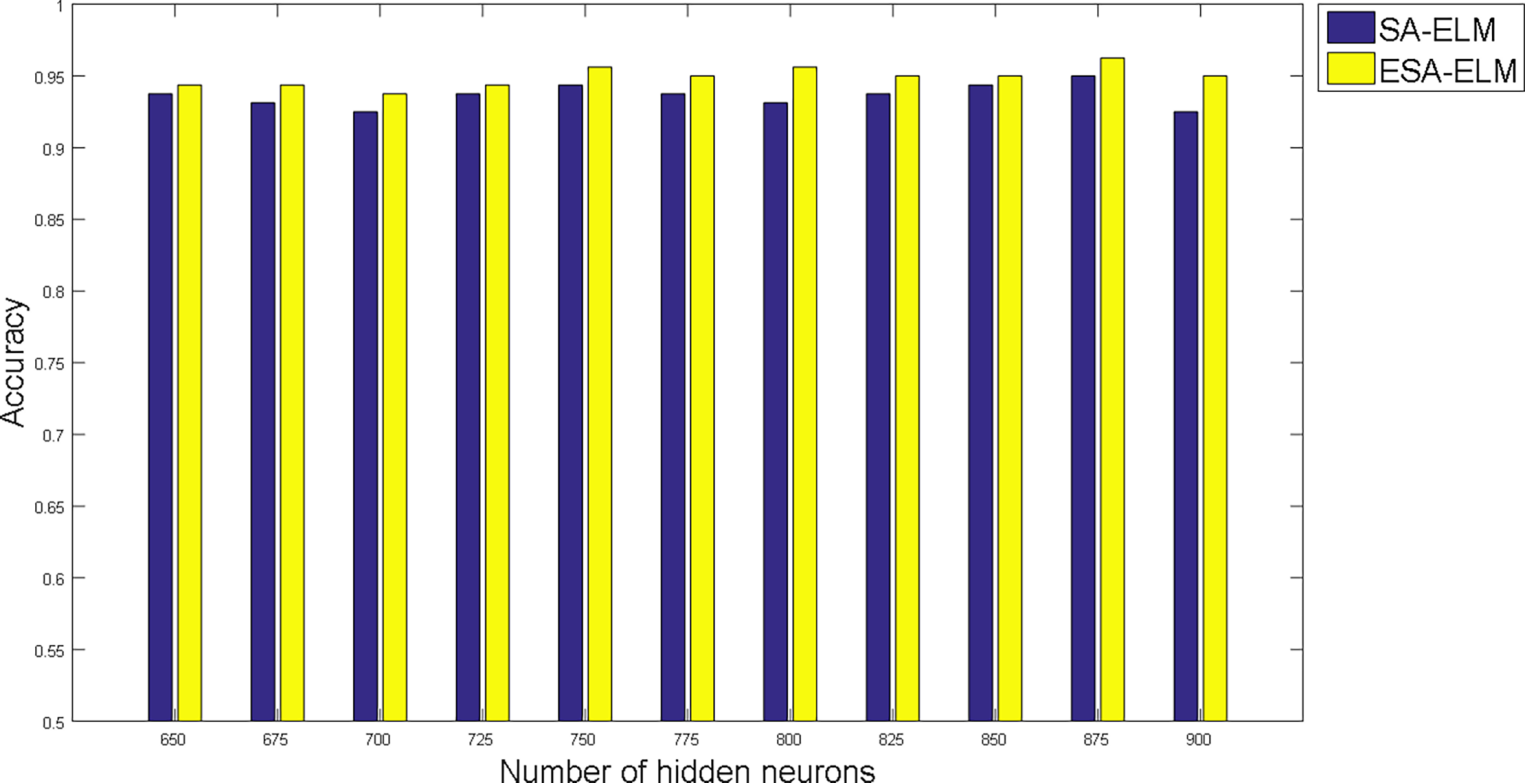
Accuracy measurement of the ESA-ELM and the SA-ELM benchmark.

**Fig 6:**
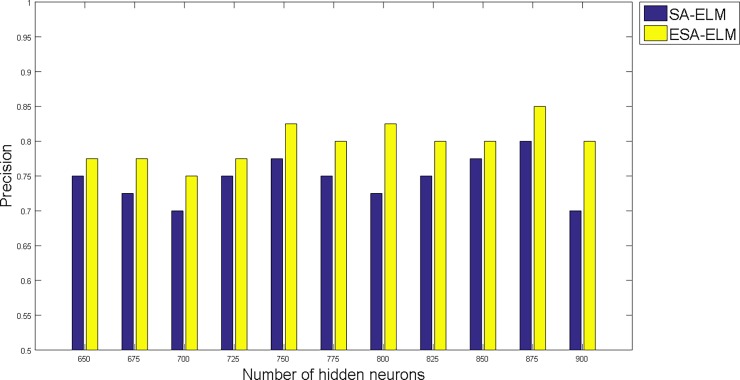
Precision measurement of the ESA-ELM and the SA-ELM benchmark.

**Fig 7:**
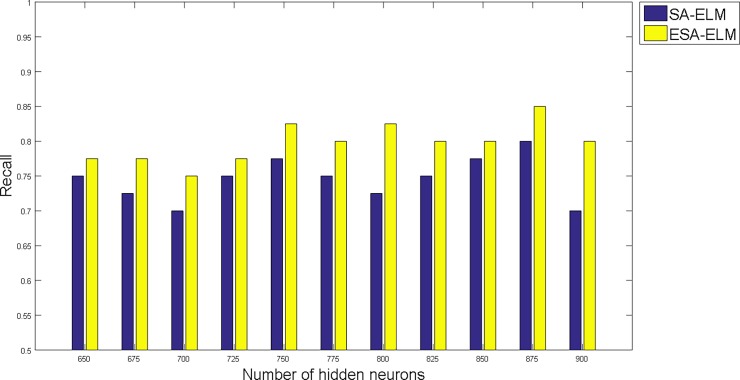
Recall measurement of the ESA-ELM and the SA-ELM benchmark.

**Fig 8:**
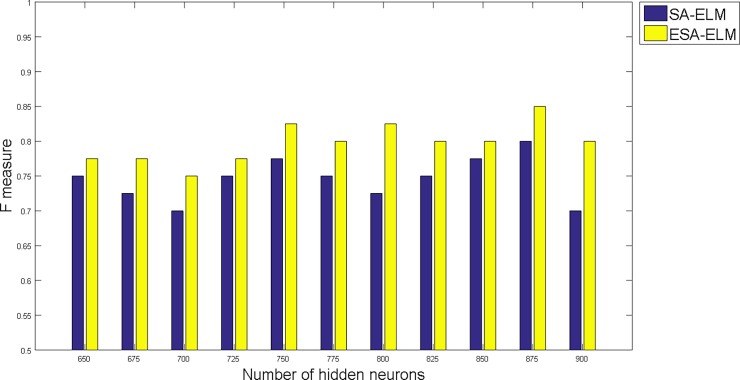
F-measure measurement of the ESA-ELM and the SA-ELM benchmark.

**Fig 9:**
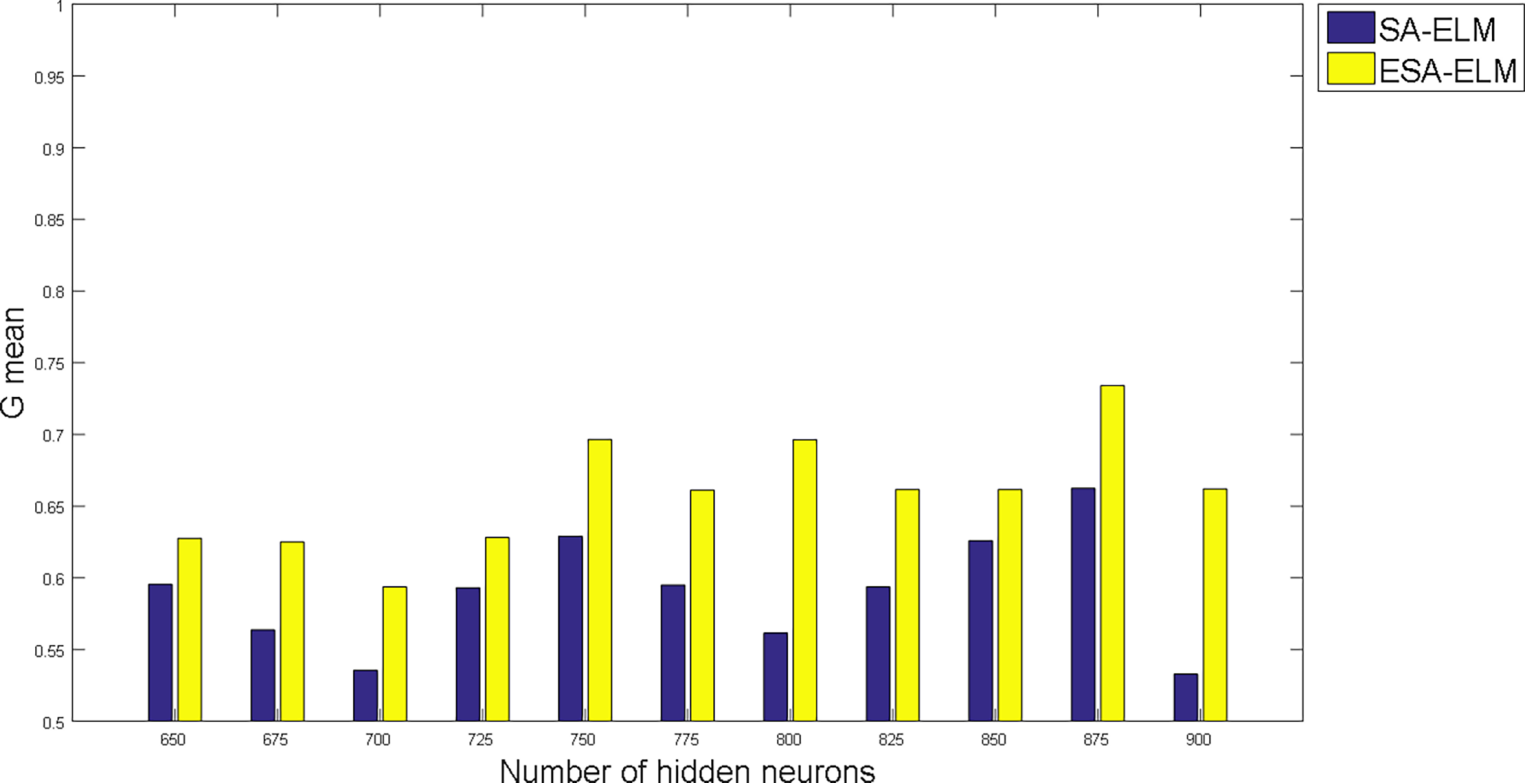
G-mean measurement of the ESA-ELM and the SA-LEM benchmark.

The following figures demonstrate the results between the SA-ELM and the ESA-ELM (Split Ratio) for all experiments conducted. The accuracy of the ESA-ELM in the range [650–900] of hidden neurones was higher than the SA-ELM benchmark. This means that the ESA-ELM performance results are much better than the SA-ELM benchmark in all iterations. “Figs 5–9” illustrate the comparative results between the SA-ELM benchmark and ESA-ELM regarding accuracy, precision, recall, F-measure and G-mean. An important observation here is that the highest accuracy was achieved for 875 neurones, refer “Fig 5”. The achieved accuracy was 96.25% for ESA-ELM and slightly lower, 95.00% for SA-ELM. Also, the obtained results of other measures for SA-ELM were; Recall 80.00%, Precision 80.00%, F-measure 80.00%, and G-mean 66.25%. While for ESA-ELM the results were; Recall 85.00%, Precision 85.00%, F-measure 85.00% and G-mean 73.41%. Tables 6 and 7 provides all the results of the Evaluation Measures through all the experiments for the SA-ELM and ESA-ELM as the following below:

**Table 6:** SA-ELM Evaluation measures through all the experiments.

	Accuracy	Precision	Recall	F-measure	G-mean
**SA-ELM 650 hidden neurons**	93.75	75.00	75.00	75.00	59.54
**SA-ELM 675 hidden neurons**	93.13	72.50	72.50	72.50	56.37
**SA-ELM 700 hidden neurons**	92.50	70.00	70.00	70.00	53.56
**SA-ELM 725 hidden neurons**	93.75	75.00	75.00	75.00	59.32
**SA-ELM 750 hidden neurons**	94.37	77.50	77.50	77.50	62.90
**SA-ELM 775 hidden neurons**	93.75	75.00	75.00	75.00	59.50
**SA-ELM 800 hidden neurons**	93.13	72.50	72.50	72.50	56.16
**SA-ELM 825 hidden neurons**	93.75	75.00	75.00	75.00	59.37
**SA-ELM 850 hidden neurons**	94.37	77.50	77.50	77.50	62.58
**SA-ELM 875 hidden neurons**	95.00	80.00	80.00	80.00	66.25
**SA-ELM 900 hidden neurons**	92.50	70.00	70.00	70.00	53.29

**Table 7:** ESA-ELM evaluation measures through all the experiments.

	Accuracy	Precision	Recall	F-measure	G-mean
**ESA-ELM 650 hidden neurons**	94.37	77.50	77.50	77.50	62.76
**ESA-ELM 675 hidden neurons**	94.37	77.50	77.50	77.50	62.50
**ESA-ELM 700 hidden neurons**	93.75	75.00	75.00	75.00	59.38
**ESA-ELM 725 hidden neurons**	94.37	77.50	77.50	77.50	62.81
**ESA-ELM 750 hidden neurons**	95.63	82.50	82.50	82.50	69.64
**ESA-ELM 775 hidden neurons**	95.00	80.00	80.00	80.00	66.11
**ESA-ELM 800 hidden neurons**	95.63	82.50	82.50	82.50	69.64
**ESA-ELM 825 hidden neurons**	95.00	80.00	80.00	80.00	66.16
**ESA-ELM 850 hidden neurons**	95.00	80.00	80.00	80.00	66.16
**ESA-ELM 875 hidden neurons**	96.25	85.00	85.00	85.00	73.41
**ESA-ELM 900 hidden neurons**	95.00	80.00	80.00	80.00	66.20

**Fig 10:**
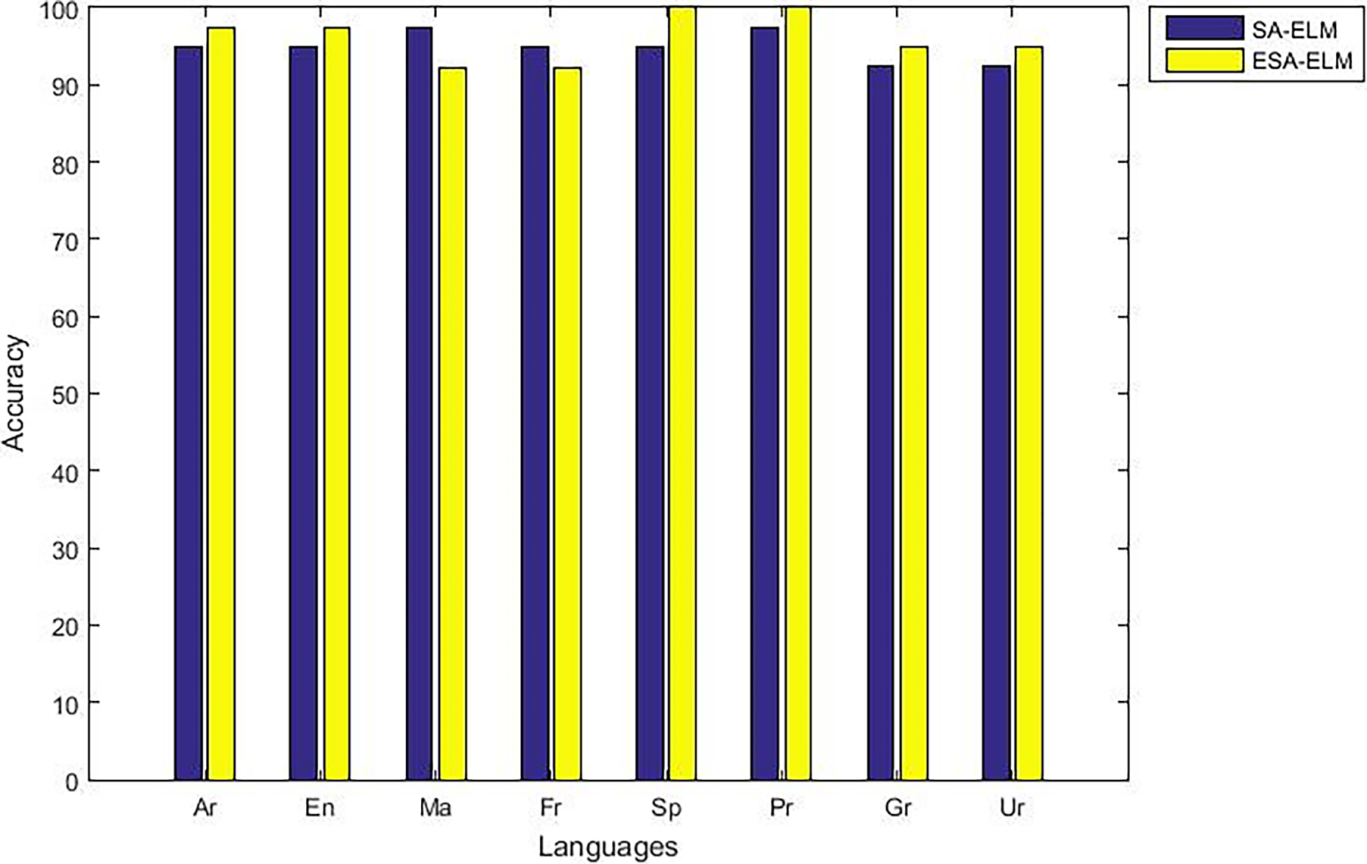
Accuracy measurement of the ESA-ELM and the SA-ELM for each language separately.

**Fig 11:**
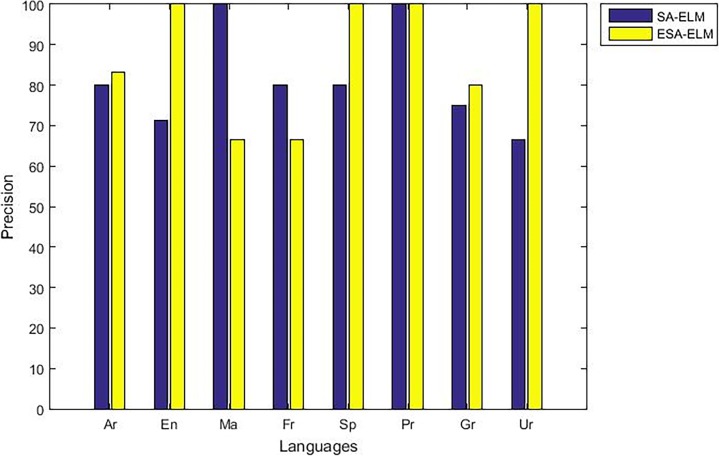
Precision measurement of the ESA-ELM and the SA-ELM for each language separately.

**Fig 12:**
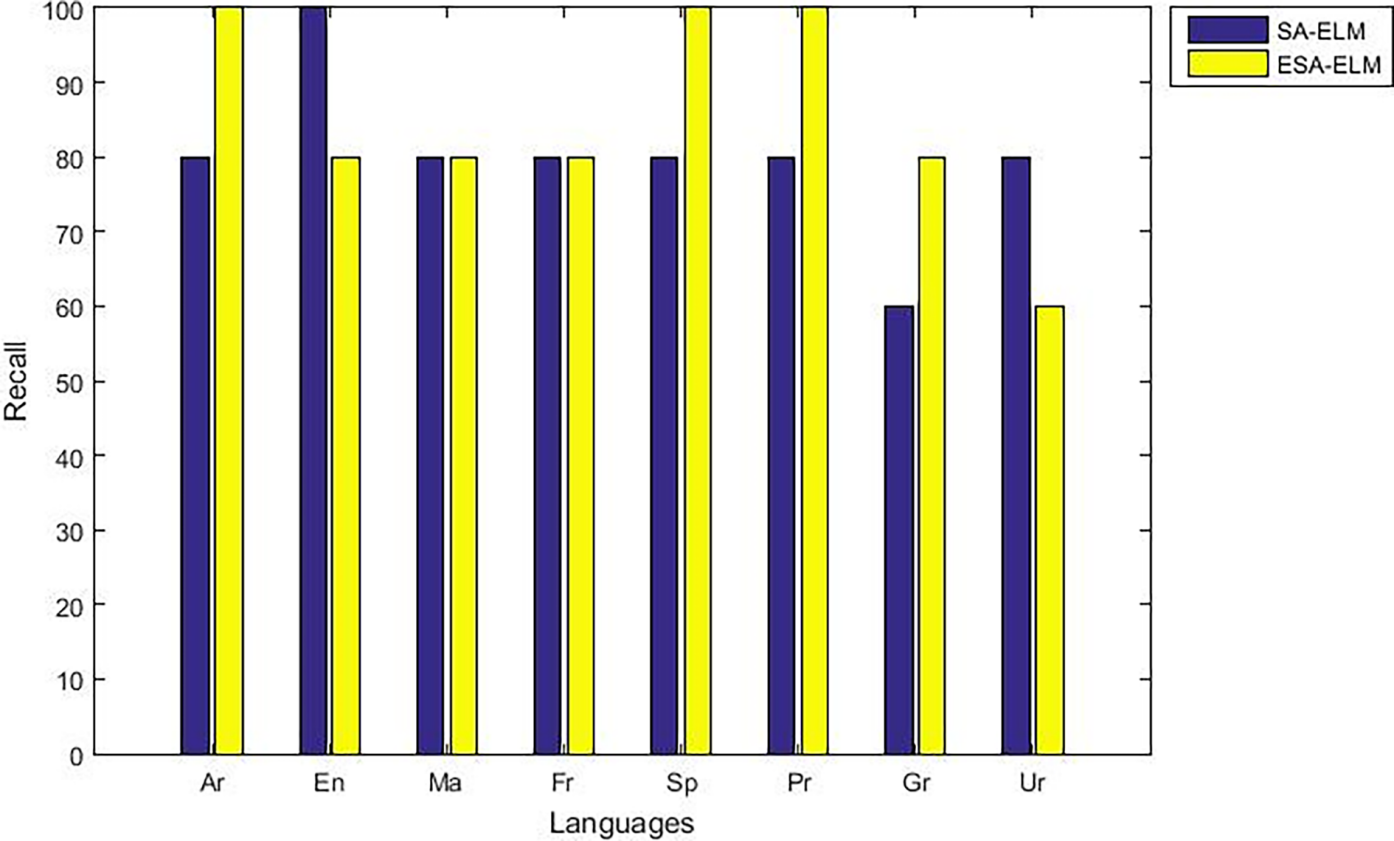
Recall measurement of the ESA-ELM and the SA-ELM for each language separately.

**Fig 13:**
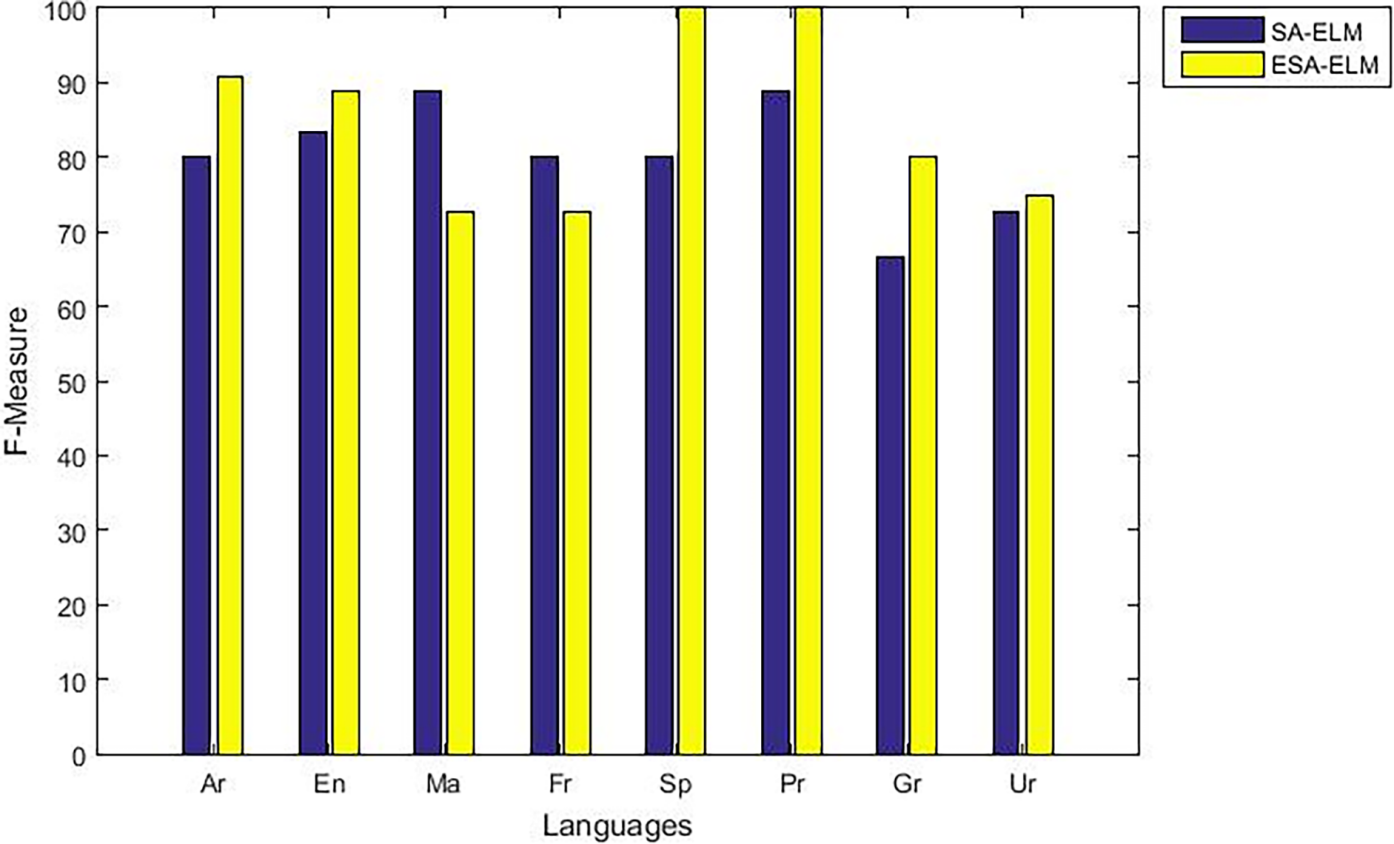
F-measure measurement of the ESA-ELM and the SA-ELM for each language separately.

**Fig 14:**
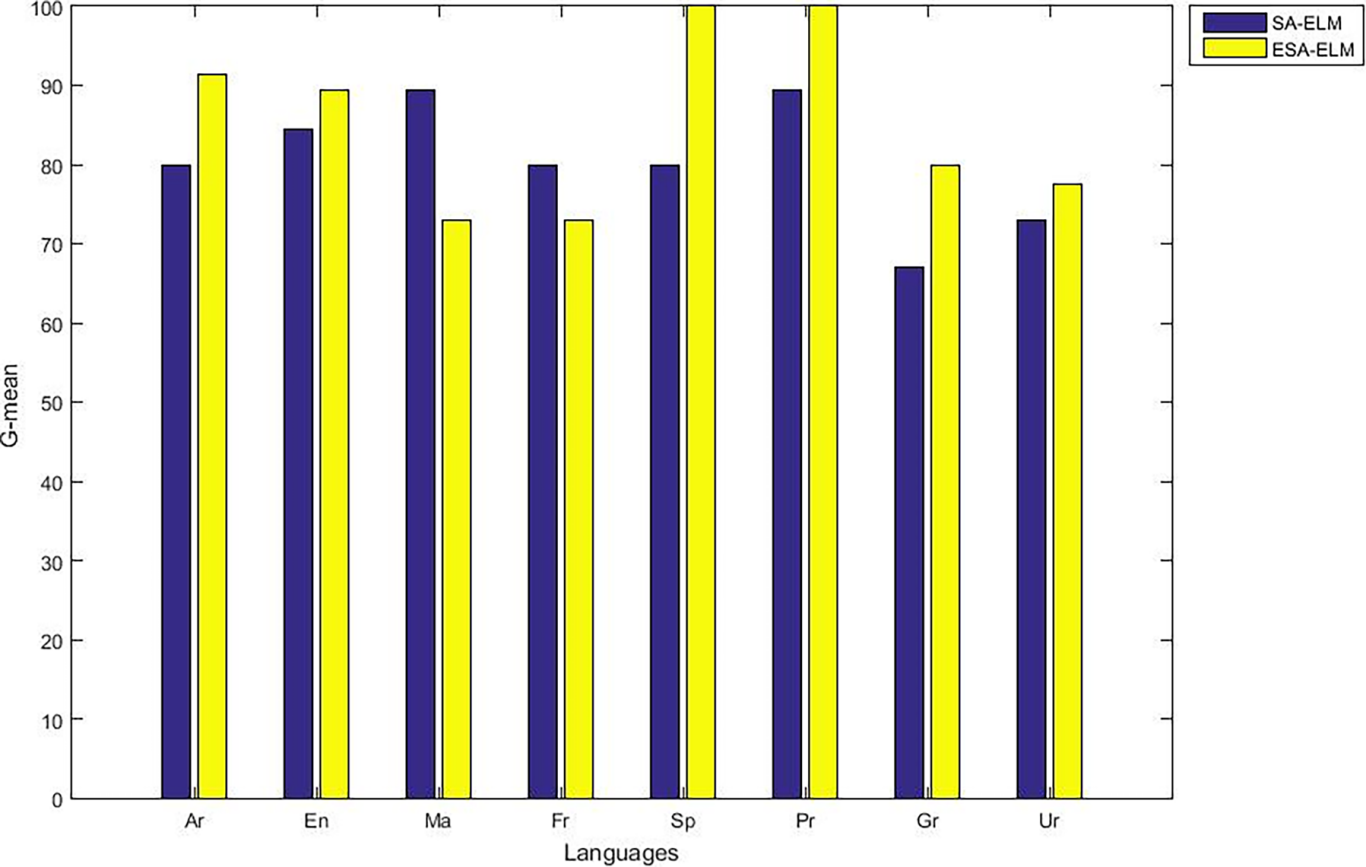
G-mean measurement of the ESA-ELM and the SA-ELM for each language separately.

As mentioned above, the highest accuracy have achieved with 875 hidden neurons therefore, “Figs 10–14” show the comparative results between the SA-ELM benchmark and ESA-ELM regarding accuracy, precision, recall, F-measure and, G-mean for each language separately with 875 hidden neurons.

**Fig 15:**
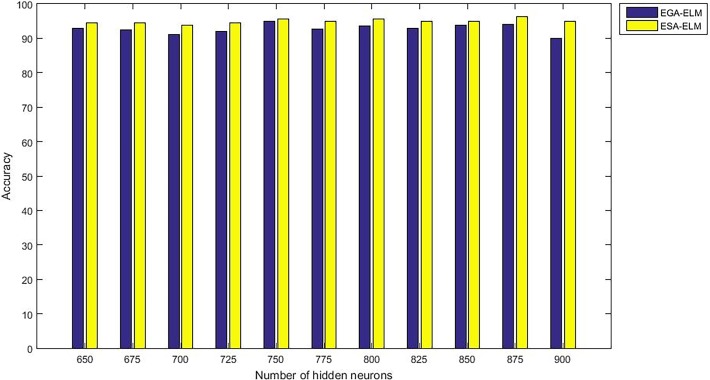
Accuracy measurement of the ESA-ELM and the EGA-ELM.

**Fig 16:**
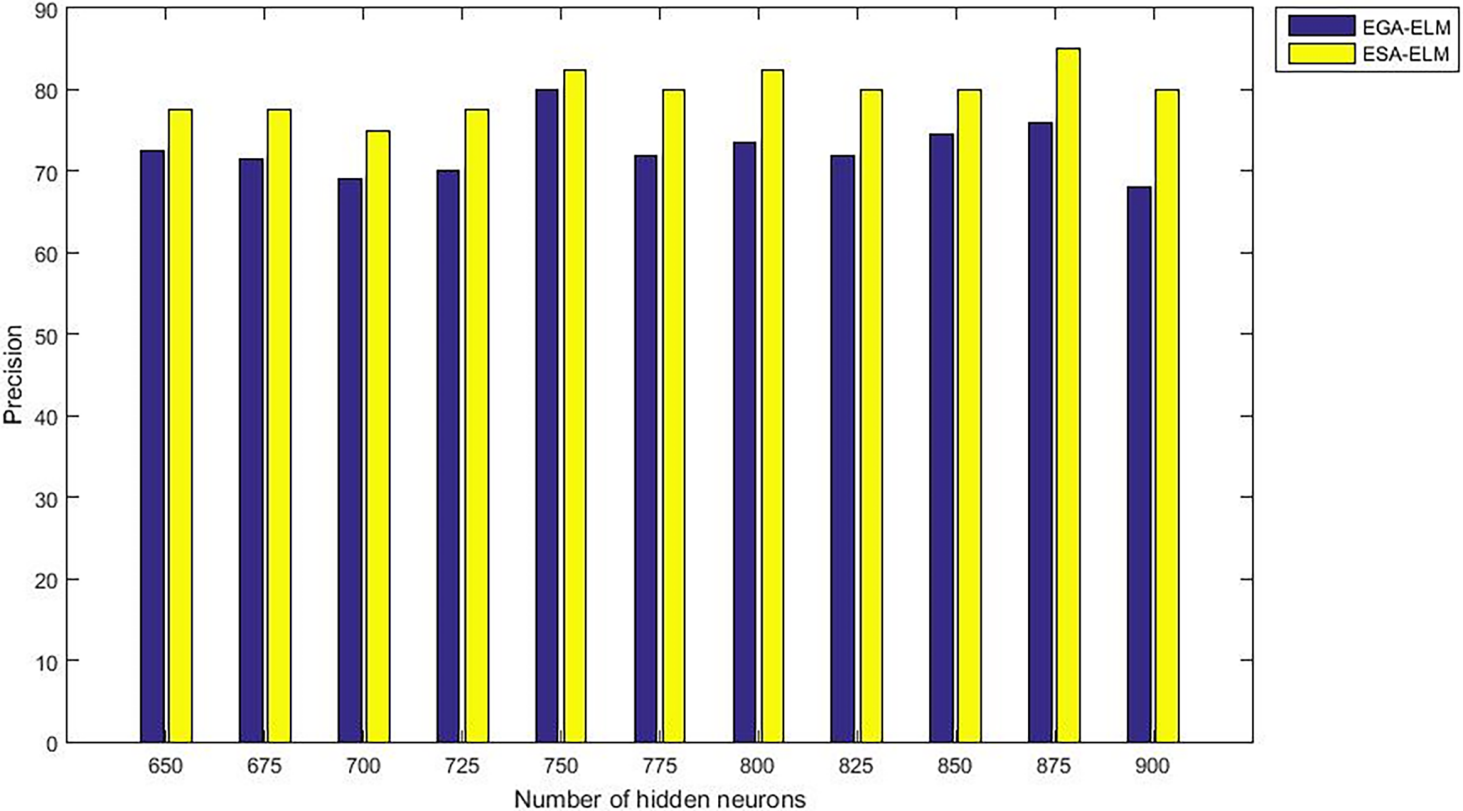
Precision measurement of the ESA-ELM and the EGA-ELM.

**Fig 17:**
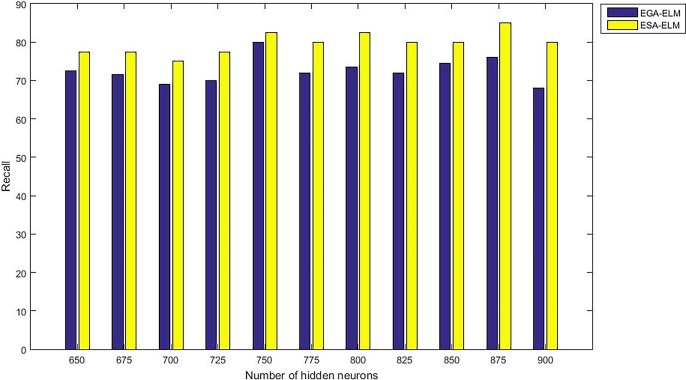
Recall measurement of the ESA-ELM and the EGA-ELM.

**Fig 18:**
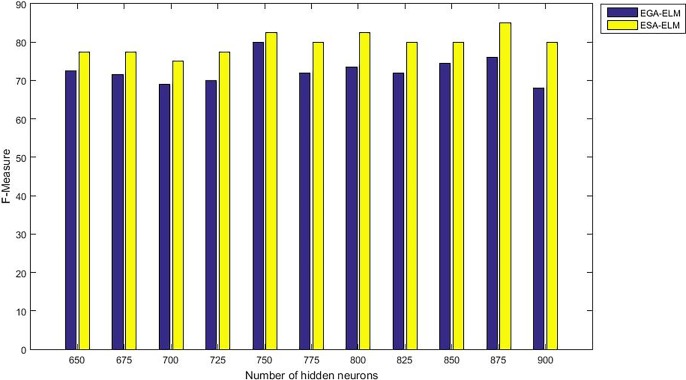
F-measure measurement of the ESA-ELM and the EGA-ELM.

**Fig 19:**
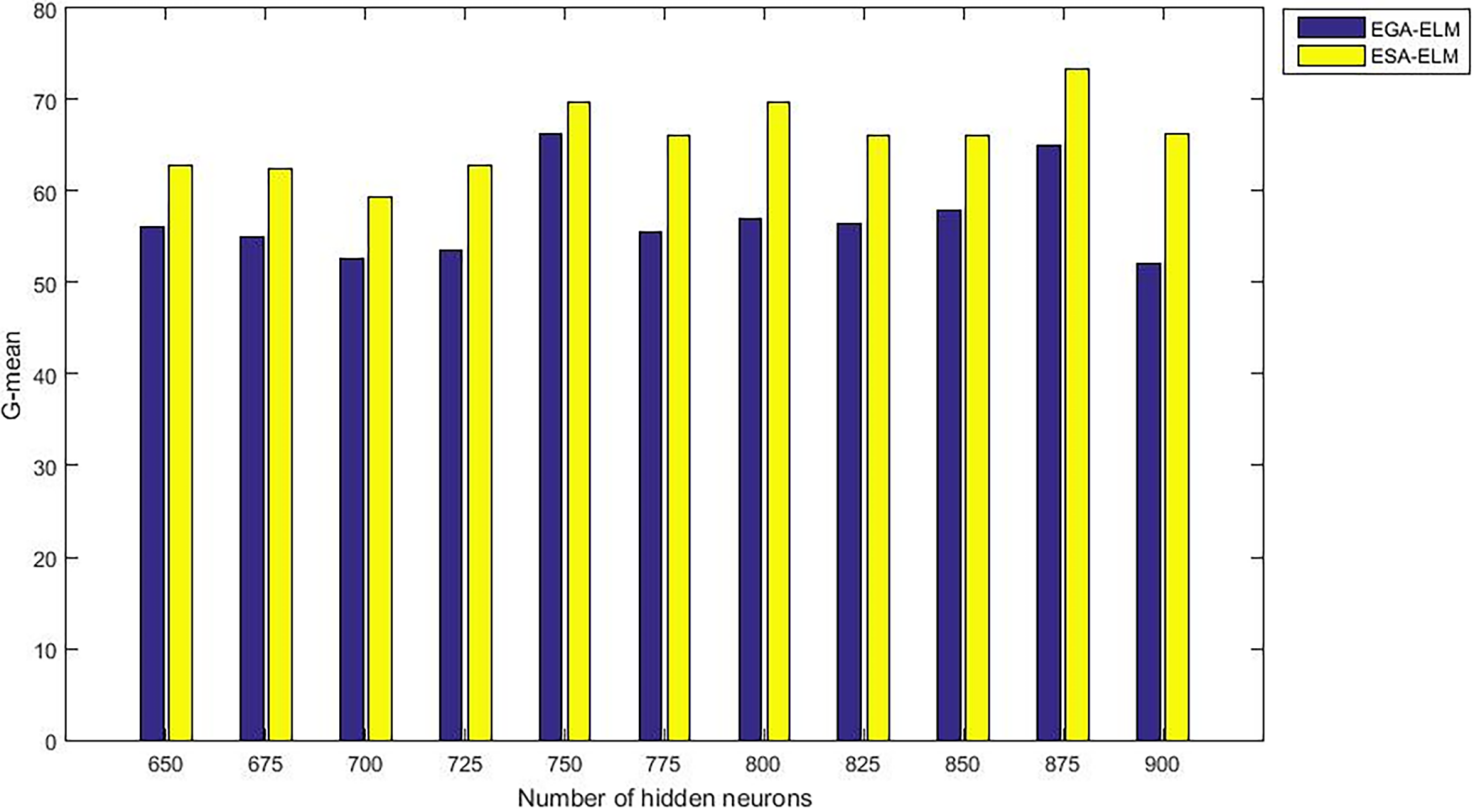
G-mean measurement of the ESA-ELM and the EGA-LEM.

Moreover, “Figs 15–19” illustrate the comparative results between the ESA-ELM and additional approach under name Elitist Genetic Algorithm Based ELM (EGA-ELM) regarding accuracy, precision, recall, F-measure, and G-mean.

## 5. Conclusion

This study enhances the existing learning model based on the ELM named as SA-ELM. The context regarding the development was to improve LID accuracy. The improvement of SA-ELM was based the optimisation approach, namely, ATLBO. ATLBO was enhanced through incorporating additional selection criteria for the searching process. The improvement was validated based on the optimisation of standard, but complex multi-variable mathematical functions and compared to the ATLBO. The EATLBO was then used in the ESA-ELM as an optimisation block for the weights of the input hidden layer neurones. The results identify the excellent (i.e. favourable) superiority of ESA-ELM compared to SA-ELM for LID. Moreover, different values of the learning model parameters were tested where the results identified the optimal parameters for learning. Following this study, the plan is to develop the LID system that can accommodate on-line execution of the feature extraction and classification while applying real-time aspects. Because only off-line LID was considered in this study. An online LID system is therefore recommended to accommodate a wider range of LID applications such as conferences, phone services, etc. Additionally, will be explored alternate optimisation methods for ELM being both cost-effective from a computational perspective and quality (integrity) from an accuracy perspective using technology. Furthermore, the front-end (features extraction) required a long time to extract the needed features thus, utilize the parallel processing can reduce the time consumption and cost greatly.

## Supporting information

S1 File LID code. (RAR)

S2 File LID dataset 2017. (ZIP)

S1 Text Provides the languages, youtube channel names, and the URLs for every single channel that we have used to collocate our dataset. (TXT)
